# Understanding family caregiving and well-being in adult chronic illness: a call for a more comprehensive perspective

**DOI:** 10.3389/fpubh.2025.1619672

**Published:** 2025-09-05

**Authors:** Bettina Schwind, Rosa Visscher, Jörg Haslbeck, Thomas Falkenstein, Christina Riewoldt, Lennert Griese, Birgit Behrisch, Sabine Metzing, Martin Nagl-Cupal

**Affiliations:** ^1^Careum School of Health, Kalaidos University of Applied Sciences, Zurich, Switzerland; ^2^Department of Medicine, Chair of Public Health and Health Services Research, Institute for Medical Information Processing, Biometry, and Epidemiology, LMU Munich, Munich, Germany; ^3^Department of Nursing Science, Faculty of Social Sciences, University of Vienna, Vienna, Austria; ^4^School of Nursing Sciences, Witten/Herdecke University, Witten, Germany; ^5^Fliedner Fachhochschule, University of Applied Sciences, Düsseldorf, Germany; ^6^School of Public Health, Bielefeld University, Bielefeld, Germany; ^7^Catholic University of Applied Social Sciences, Berlin, Germany

**Keywords:** family caregiving, family well-being, chronic illness, health equity, interdisciplinarity

## Abstract

The rising burden of chronic illness, driven by increased life expectancy and an aging population, has amplified the demand for family caregiving and research thereof to assure the well-being of families in the future. Addressing these challenges requires an evaluation of the existing gaps in research on family caregiving and well-being in adult chronic illness. In order to achieve this, seven scholars from various academic disciplines who are researching this topic in Austria, Germany, and Switzerland convened for a two-day workshop in 2024. Discussions were complemented by a set of initial literature searches. The workshop revealed that studies documenting the burdens of informal caregiving have tended to overlook the broader family and social contexts, as well as the well-being of families as a whole, by focusing on single perspectives and improvement of disease management. Thereby, current research fails to address the diverse needs of all family members involved and often neglects intertwined factors like gender, socioeconomic status, and access to the formal health and care system. This results in gaps in how these intertwined factors influence family caregiving and well-being. We propose a more comprehensive, interdisciplinary investigation of family caregiving and well-being in future studies. Further scientific consideration is needed to adequately address the structural and procedural barriers to (in)formal support for families. Understanding the real-life complexities of caregiving can contribute to bridging gaps in research and practice, while promoting family-centered approaches to contribute to health equity. Research and practice recommendations are provided.

## 1 Introduction

The burden of chronic diseases, defined as health conditions that persist and necessitate continuous management over an extended period (1), is increasing in Europe due to improved life expectancy and demographic changes toward an aging population (2, 3). Family members provide substantial support to chronic ill adults, understood as informal care that is characterized by a lack of formal training or financial compensation (4). This informal care provided by families is an important cornerstone of health care (5, 6). Due to the predicted shoratage of health professionals, there will be an even greater need for family members to five care in the future (5, 7–10). However, families are constantly under change. For example, family members are more geographically dispersed which leads to shifts in the set-up of households and what it means to be a family (11, 12). This highlights that what constitutes a family is bound to social change (12), but affecting who is considered to need (formal) support and thus a matter of (intergenerational) health and equity concerns (13, 14). As family caregiving of chronically ill individuals is of critical importance to the functioning of our communities and societies (15, 16), it is of the utmost importance to better understand the complex issues at stake to be best prepared for the challenges ahead.

In light of the complex interplay between adult chronic illness, family caregiving and wellbeing, it is imperative to undertake a comprehensive evaluation of the existing research gaps from an interdisciplinary perspective. This is warranted by the recognition that diverse academic disciplines may approach this subject from various perspectives (17), thereby encouraging innovation, creativity, productivity and overall research success (17, 18). To achieve this, a two-day workshop on “Chronic illness and family over time”, funded by the Swiss National Science Foundation (222885), was held in February 2024 in Zurich, Switzerland. (see the agenda in Supplementary material S1). The workshop included seven scholars (see authors) from diverse academic backgrounds, including public health, nursing, education, disability studies, sports, social sciences, and gender studies to enhance reflexive problem understanding. The scholars came from research institutions in Austria, Germany, and Switzerland and were of different genders, ages and research seniority to ensure a diversity in perspectives. In preparation for the workshop, various initial literature searches were conducted to support the workshop discussions (details on the review process are provided Supplemental material S2).

This perspective article focuses exclusively on presenting the workshop discussions on current findings and research gaps in relation to family caregiving and well-being in adult chronic illness. It is important to acknowledge that both terms - family caregiving and well-being - are very broad and lack precise definition, instead respresenting social and therefore deiscipline-specific constructs that require careful reflection (19). Given this inherent uncertainty and the breadth of workshop discussions, which included theoretical, methodological and ethical considerations, this article is pertaining to family caregiving and well-being in adult chronic illness only to ensure coherence and actionable insights. This focus enables coherence on aspects that were rich and the insights to be actionable. While broader reflections on theoretical and ethical aspects were invaluable, reporting them would risk to dilute clarity. Aware of this, we will first describe the fragmented evidence on family caregiving in chronic illness from the perspective of health and social sciences. Second, we will present how family well-being in chronic illness is assessed and discuss its strengths and limitations. Third, we will stimulate a discussion on the implications for research and practice, providing recommendations for future directions. Therewith, the authors of this perspective article offer several contributions to the current field of (applied science) research with regard to family caregiving and well-being in chronic illness to ensure the sustainable and equitable promotion of family-centered care to support family well-being.

## 2 Family caregiving in chronic illness

The health sciences have demonstrated a particular interest in family caregivers as proxies for ill people with the aim of providing support and managing illness trajectories (20). The challenges and burdens experienced when providing care for a family member with a chronic illness have been the subject of extensive research and documentation (6, 21, 22). Studies have documented the negative impact of caregiving on physical, emotional, financial, relational, educational, occupational, recreational, and social aspects (23, 24), but have also identified potential stress-relieving effects (25, 26). This research has led to a broad consensus that family caregivers need support, based on the assumption that a causal relationship exists between caregiving responsibilities and overburden (27). However, this view overlooks the diversity of skills and competencies of family caregivers (28). For instance, family members have demonstrated the ability to negotiate different caregiving roles and responsibilities within various family networks, a process that is constantly evolving (29). Health literacy has been reported as being unevenly distributed across families’ social networks and used as a resource when and where needed (30). Therefore, not all family caregivers possess the same competencies and management styles to cope with different strains and thus may differ in their support needs. Moreover, empirical studies on caregiving in families tend to focus on single perspectives, such as those of adult family caregivers (21), of children and young people as family caregivers (31), or of dyadic relationships between spouses (32, 33), thereby almost decontextualizing caregiving from the family and social context (23, 33, 34). Such studies overlook the importance of family relationships and the ways in which they are part of life courses and distinct biographies, while individuals may be both caregivers and care-receivers at the same time (35).

From a social science perspective, the family, as an intergenerational community has demonstrated resilience despite all changes in the context of illness and caregiving challenges (36) and is still considered as a ‘hidden health system’ (37). Women up until now shoulder the primary responsibility of caregiving for chronic illness in the family (5, 38, 39). While men also give care, they apparently face higher levels of stigma (40). At the same time, families are diversifying, with increases in one-child households and patchwork families (35). These dynamic changes in family caregiving suggest that more comprehensive approaches in chronic illness may be needed (41). This is particularly true given the intricate interweaving of chronic illness into family life, with its attendant influence on and influence from gendered family norms, ambivalences of dependence and independence, and the tension between collective “we” identities and individual autonomy (42–44). In addition, the role of the family in caregiving is shaped by economic, educational, social, and health system contexts (36) and involves interactions between informal and formal care systems (45). These dynamics are further influenced by factors such as socioeconomic status, educational attainment, migration-related barriers, and racial discrimination (46, 47), which require further understanding concerning family caregiving.

## 3 Family well-being in chronic illness

Despite the absence of a universially accepted definition of family well-being in the health sciences, its is understood to be a multidimensional concept encompassing physical, social, economic, and psychological dimensions (48–50). It is often explored through related concepts such as family functioning, resilience, health, and flourishing (49); it is also treated as a dimension of family functioning (51), which is considered to be a cornerstone for assessing the family quality of life (QoL) (52). There is an overall overlap between the different concepts, as for example between QoL and well-being, with the latter being typically used more broadly and holistically (53).

Due to the multidimensional nature of family well-being, various tools have been used to measure it in the context of chronic illness, such as those assessing family QoL (48) or family functioning (54). Although these tools differ in focus, a common feature is that they primarily assess the individual perspective of either the ill person, the primary caregiver, or a family member. However, it is questionable whether the perspective of one member can capture the complexity of a family’s overall well-being (55).

A growing body of literature demonstrates the reciprocal relationship between individual and family well-being (56, 57), highlighting the need for approaches that consider the family as a whole (48, 58, 59). However, tools that capture the perspectives of multiple family members remain limited (60–62). Those available are predominantly validated for traditional “married with child” families and often lack the capacity to provide an overall family score. Furthermore, as recent reviews have highlighted, most measurement tools have been used in studies with participants who were white or presumed to be white (54). As a result, little is known about family well-being and functioning in families from diverse backgrounds.

Moreover, most family well-being measures originate from the late 1970s and early 1990s, drawing primarily on the McMaster Model or the process Model of Family Functioning as well as findings from family interviews (60, 62, 63). These early tools, listed in Table S3 in the Supplementary material, were intended for screening families in need of support and as research instruments. Scholars typically achieved validation by comparing responses from one family member with findings from interviews. The earlier tools show a focus on family functioning, covering dimensions such as problem-solving, communication, roles, affective response, affective involvement, behavior control, as well as values and norms, but not on how such aspects as, e.g., roles are viewed from different perspectives. Subsequent tools narrow their perspective to specific aspects of the original measures (64, 65) or are more closely aligned with QoL frameworks, incorporating domains such as emotional well-being, physical/material/financial well-being, and support (66, 67). Over time, many family well-being assessment tools have emerged, each varying slightly in focus and domain coverage. This proliferation has led to review articles (48, 54, 61) and initiatives^1^ aimed at cataloging and summarizing existing tools. Despite these efforts, a lack of consensus remains, as single tools only cover partial aspects of family well-being (Table 1).

**Table 1:** Overview of the different family well-being assessment tools.

Concept	Scale	Domains	Number of items	Target populations	Available languages	Year
Family functioning	Family Environment Scale (FES)	1. Family relationship, 2. Personal growth, 3. System maintenance and change	90	Adult(s) and/or child(ren) from 11 years onwards	>22 languages, incl. Eng, Chi, Jap, Mal, Spa, Por, Gre, Heb	1974
	Family APGAR Index: Family Adaptation, Partnership, Growth, Affection, and Resolve	1. Adaptation, 2. Partnership, 3. Growth, 4. Affection, 5. Resolve	5	Adult or child from the age of 10 onwards, for families with chronically ill person	Eng, Chi, Jap, Spa	1979
	Family Assessment Device (FAD)	1. Problem solving, 2. communication, 3. Affective responsiveness, 4. Affective involvement, 5. Behavior control, 6. General functioning	60	Adult or child from 12 years onwards, for families with chronically ill person	Eng, Ger, Fre, Chi, Gre, Hindi, Ice, Ita, Por, Spa, Swe, Tur	1983
	Family Relationship Index (FRI)	1. Family cohesion, 2. Family expressiveness, 3. Family conflict	12	Adult(s) and/or child(ren) from 11 years onwards, for families with chronically ill person, with and without child(ren), for couples as well as single parents	(see FES)	1983
	Assessment of Strategies in Families-Effectiveness (ASF-E)	1. System maintenance, 2. System change, 3. Coherence, 4. Individuality	18	One person representing the family, unclear if this could be a child	Eng, Ger	1991
	Family Functioning Family Health and Social Support (FAFHES)	1. Affect, 2. Affirmation, 3. Concrete aid	20	Designed for caregiver, with and without child(ren), living in same household	Eng, Ger, Per	2002
	Brief Assessment of Family Functioning Scale (BAFFS)	1. General functioning	3	Adult caregiver with or without child(ren), living in same household	(see FAD)	2019
Family QoL	Beach Family Quality of Life Scale (FQOL)	1. Family Interaction, 2. Parenting, 3. Emotional Well-being, 4. Physical Well-being / Material Well-being, 5. Disability-Related Support.	25	Adult in family with child(ren) with or without disability, also possible for a parent with a disabilty	Eng, Fre, Spa	2006
	(Canadian) Family Quality of Life survey	1. Health of the family, 2. Financial well-being, 3. Family relationships, 4. Support from other people, 5. Support from disability-related services, 6. Influence of values, 7. Careers and preparing for careers, 8. Leisure and recreation, 9. Community involvement	54	Main adult caregiver with child(ren) with or without intellectual and/or developmental disabilities	Eng, Ger, Bos, Chi, Dut, Fle, Far, Fre, Ita, Jap, Mal, Pol, Por, Slo, Spa, Tel	2006
	Family Reported Outcome Measure (FROM-16)	1. Emotional, 2. Personal and social	16	Adult with (chronic) ill family member of any age	>30 translations, incl. Eng, Ger	2014
Family Health	Family Health Scale	1. social and emotional health processes, 2. family healthy lifestyle, 3. family health resources, 4. family external social supports	32 (short version 10)	Adult(s) in heterosexual relationship with child(ren)	Eng, Chi, Por	2020

Eng, Englisch; Chi, Chinese; Jap, Japanese; Mal, Malay; Spa, Spanish; Por, Portuguese; Gre, Greek; Heb, Hebrew; Ger, German; Fre, French; Per, Persian; Ice, Icelandic; Ita, Italian; Swe, Swedish; Tur, Turkish; Bos, Bosnich; Dut, Dutch; Fle, Flemish; Far, Farsi; Pol, Polish; Slo, Slovenian; Tel, Telugu. Further details of the scales mentioned are given in the, see Supplementary Table S3.

## 4 Discussion

Our interdisciplinary workshop catalyzed an engaging and mutual beneficial learning experience about strengthening the evidence base for family caregiving and well-being in chronic illness. It brought together diverse perspectives and bodies of knowledge from the health and social sciences, highlighting the need for an interdisciplinary approach that acknowledges the complexity and heterogeneity of family caregiving realities across different health and care systems.

A substantial proportion of research has focused on family caregivers of individuals with chronic illnesses. However, the broader family and social context in which these caregivers operate has received comparatively less attention than the study of their relationships (23, 33, 34). Consequently, there is an urgent need for a more comprehensive understanding of family well-being, one that transcends the confines of a single perspective as a proxy. This calls for acknowledging the complexities, diversity, and relational reciprocity inherent in caregiving experiences across diverse families and among their members, while recognizing that family caregiving is inextricably intertwined with the formal care services of the broader welfare system (68–71). This type of evidence is paramount for developing effective support strategies tailored to the needs of family members most in need of support. Only thereby, can we contribute to the overall well-being of the family and address social inequalities within the broader health and care systems. Based on this, the following research and practice recommendations are considered of utmost importance in tackling the challenges ahead for our aging societies. Research and practice recommendations are:

1. Call for interdisciplinary research approaches that integrate health sciences, social sciences, and family studies. Emphasize the importance of intersectionality to explore how family caregiving and well-being in chronic illness is shaped by diverse factors such as gender or socioeconomic status (72).
2. Encourage (longitudinal) research that considers a lifespan perspective and/or the complex, lived realities of diverse families that do not necessarily follow linear causality (54). Focus future research on exploring the interrelated experiences of family members as a unit, rather than in isolation, highlighting the necessity of a systems-thinking lens to examine the complexity of informal-formal caregiving interactions across different health and social systems (11). Such research perspectives would allow for a deeper understanding of the diversity and heterogeneity of families, including their ambivalences (35), while considering the specifics of the health and care system (45, 69).
3. Greater participation of individuals/families in research, practice, and policy so that they can influence agendas in ways that are meaningful to them (73, 74), for example through priority setting (73), while accounting for the diversity of families and those in vulnerable situations.
4. Conduct a concept analysis (75) to develop a conceptual understanding of family well-being. This will lay the groundwork for the development of comprehensive tools that assess family well-being with scales that account for the diverse perspectives and meanings. Such scales could be a valuable addition to a nursing practice’s toolkit for identifying the most vulnerable family situations and members for support interventions.
5. Stronger acknowledgement and integration of a family-centered approach into primary care services and coordination of support services from health to social care.
6. Introduce policy frameworks that support comprehensive family-centered care interventions, which consider the needs of all: the ill person, informal caregiver, and the entire family as a unit, to mitigate health inequalities.
7. Ultimately, such a more comprehensive interdisciplinary approach toward research, policy and practice would enable a more nuanced understanding, which would support the development of targeted and effective interventions for informal caregiving families and improve their overall well-being.

This research perspective integrates evidence, expertise, and knowledge from various disciplines. However, it has some limitations that must be acknowledged. The workshop format encouraged reflective thinking and collaborative learning among scholars of diverse disciplines, ages, and genders. The initial literature searches provided a foundation for the discussions by identifying gaps in the existing literature. Because these searches were not systematic, we may not have considered all available evidence (76), and the discussions may have been influenced by group and power dynamics (77). Nevertheless, the interdisciplinary workshop format enabled us to contextualize the identified evidence within various scholarly traditions (78). This provided a more comprehensive overview than would have been possible otherwise (78), allowing us to generate ideas for innovating research on the matter openly. The discussions also addressed theoretical underpinnings and ethical considerations of family caregiving in adult chronic illness. When discussing ethical aspects in family research from an interdisciplinary perspecptive, divergent epistemic frames of references were encountered (79), with the emerging complexity extending beyond the scope of this article.

## 5 Conclusion

The complexities of family well-being in the context of chronic illness are characterized by a fragmentation of focus, with a prevailing emphasis on the perspectives of caregivers within the field of health sciences, who are considered proxies for the chronically ill person. It is imperative to recognize the necessity of an interdisciplinary and more comprehensive approach to understand the complexity of caregiving for chronically ill people within diverse and changing families, as this is the only way to ensure health equity in the context of an aging society undergoing social change.

## Data Availability

The original contributions presented in the study are included in the article/Supplementary material, further inquiries can be directed to the corresponding author/s.

## Footnotes

^1^https://healthychild.ucla.edu/programs/life-course-translational-research-network/resources/family-functioning-measures; https://internationalfamilynursing.org/resources-for-family-nursing/research/measurement-resources/; https://fam-net.org/instruments-family/page/3/?product_cat=fwb.
