# Supplementary material for: Understanding family caregiving and well-being in adult chronic illness: a call for a more comprehensive perspective
Source: Front Public Health. 2025 Sep 5;13:1619672. doi: 10.3389/fpubh.2025.1619672 (PMC12446232; doi:10.3389/fpubh.2025.1619672)
Supplement: Supplementary file 1 [file Table_1.docx]

Supplementary Material

The supplementary material includes:

- S1 Workshop agenda Page 2
- S2 Brief summaries of the initial literature searches conducted in preparation for the workshop Page 5
  - Table S2.1: Overview of the search strategies for the initial literature searches Page 7
  - Table S2.2: Overview of the included studies from the initial literature searches Page 10
  - References from the initial literature searches Page 28
- Table S3: Overview of instruments to measure family well-being Page 34

- **S1 Workshop agenda**

Funding: SNF IZSEZ0 222885

Location: Careum School of Health (CHG), Zürich (CH),

Moderation: Prof. Dr. Jörg Haslbeck & Dr. Bettina Schwind

**Monday, 26. February 2024**

| **Time** | **Topic** | **Description** |
| --- | --- | --- |
| 08:30-09:30 | 1. Arrival/Welcome | Short welcome to all attendees.  Presented by: Dr. Stefan Spycher, CEO Careum  Ice-breaker: what are your expectations for this 2-day workshop? |
| 09:30-10:00 | 2.1 Presentation/Discussion | “Chronic illness and family dynamics in Austria”  Presented by: Prof. Dr. Martin Nagl-Cupal, University of Vienna |
| 10:00-10:30 | 2.2 Presentation/Discussion | “Chronic illness and family – focus Switzerland”  Presented by: Prof. Dr. Jörg Haslbeck, CHG |
| 10:30-11:00 | Break |  |
| 11:00-11:30 | 2.3 Presentation/Discussion | “Young Carers research at Witten/Herdecke University in Germany”  Presented by: Prof. Dr. Sabine Metzing, Witten/Herdecke University |
| 11:30-12:00 | 2.4 Presentation/Discussion | “Relationship and negotiation processes within families with chronic illness in Germany from the perspective of sociology / disability studies”  Presented by: Prof. Dr. Birgit Behrisch, Catholic University of Applied Sciences |
| 12:00-13:30 | Break |  |
| 13:30-16:00 | 3. Results & Discussion to initial Literature Searches  4.1 Introduction to Concept Mapping | “Results of the initial literature searches” (see S2)  Presented by: Dr. Lennert Griese, Bielefeld University; Thomas Falkenstein, University of Vienna; Christina Riehwoldt, Witten/Herdecke University  Discussion of the identified research gaps.  “Introduced to the methodology of concept mapping.”  Presented by the moderators  Each participant was given sticky notes and a pen and was tasked with writing down possible research gaps. Afterwards sticky notes were grouped into overarching topics. |
| 16:00-16:30 | Break |  |
| 16:30-17:00 | Wrap-up | Summarizing the results of Workshop Day 1 |

**Tuesday, 27. February 2024**

| **Time** | **Topic** | **Description** |
| --- | --- | --- |
| 09:00-12:00 | 4.2 Concept Mapping - Group Work | Based on the outcomes of the previous day, the following definitions were discussed: Family/relatives, chronic illness. Attendees were divided into two groups:   - Group 1 - focus on “Theoretical underpinnings & ethical considerations” - Group 2 – focus on “Methodological approaches & ethical considerations”   Each group reviewed the identified literature from the initial literature searches that were presented during day 1. Group 1 extracted possible theoretical underpinnings, while Group 2 extracted possible methodological approaches. Relevant keywords were written down on sticky notes and grouped into overarching themes. |
| 12:00-13:00 | 4.3 Concept Mapping - Plenum | Each group briefly presented its main outcomes.  Afterwards, each participant received six stickers: three yellow and three black. With the yellow stickers, attendees could indicate which sticky notes had the most practical relevance. With the black sticky notes, they could indicate the sticky notes with the highest scientific relevance. |
| 13:00-14:30 | Break |  |
| 14:30-16:45 | 4.4 Round Table Discussion and Prioritization | The highest ranked sticky notes were discussed and formulated into research recommendations. |
| 16:45-17:00 | Break |  |
| 17:00-18:00 | 5.Wrap-up / Closing | Reflection on the workshop results and possible next joined steps  Closing remarks  Presented by: Prof. Dr. Jacqueline Martin, CEO CHG |

- **S2 Brief summaries of the initial literature searches**

In preparation of the workshop, four initial literature searches were conducted to identify research gaps related to the topic of "family caregiving and chronic illness". The approach of initial literature searches was exploratory and chosen to enable a quick overview of the current research landscape, considering the team’s limited timeframe and resources. Each search, screening, and data extraction was conducted by one scientist from one of the four involved institutions. Meetings between these scientists took place to decide on the focus of the searches, optimize the search strategy, define in- and exclusion criteria, and discuss outcomes and how to present these at the workshop for initiating discussions. The focuses of the four initial searches were discussed within the meetings and chosen to limit the screening workload of each person, while maintaining relevance and insight for the specific institution conducting the search so as to keep everyone involved. Therefore, the initial search conducted by researchers of the Careum School of Health from had a country-specific focus, as they were specifically interested to map the current evidence base for Switzerland. In consideration of the necessity of ensuring the manageability of retrievals whilst providing a well-founded overview, the initial search period was designated as the preceding decade. In preparation for the workshop, all initial searches were conducted at the end of 2023, thus comprising articles published between 2013 and 2023. Nevertheless, the search with a focus on health literacy was inadvertently not confined to the preceding decade, as demonstrated in Table S2.1 below. Following deliberation, it was determined that the summary findings from outside this time period would not be retrospectively deleted.

The main takeaways from the initial searches are summarized below. Table S2.1 details the search strategies, including the in- and exclusion criteria, and Table S2.2 summarizes all articles included and used during the workshop discussions.

During the workshop, the main findings of the initial searches were presented. Additionally, the full texts of the included articles were shared with the workshop attendees and used to extract "theoretical underpinnings" and "methodological approaches", as described in the workshop agenda topic 3.

Focus: Chronic illness & family caregiving in daily life:

This result offers valuable insight into life with a chronic illness and the experiences of affected family members, such as environmental, social, and gender aspects, and the measuring of the experience of living with chronic illness:

- Joo and Liu (2022) describe in their study on the transition from hospital to home care that family members play a pivotal role in caring for chronic illnesses at home. Park and Schumacher (2014) point out that the caregiver-recipient relationship is characterized by mutual interaction. Vick and Wolf (2021) and Whitehead et al. (2018) further stress the importance of family support and engagement in caring for a family member with a chronic illness. Marin-Maicas et al. (2021) explore the complex, nonlinear process of living with a long-term illness from a family's perspective. These processes are characterized by acceptance, coping, self-management, integration, and adaptation, which illustrate the complexity of living with a chronic illness. With this in mind, Dugglebey et al. (2021) emphasize the importance of hope for family caregivers and the positive outcomes associated with it.
- In Addition, Eifert et al. (2016) examine the aging baby boomer generation from a life course perspective, offering insights into the evolving trends in chronic illness. According to Bueno and Chase (2023) and Fee et al. (2020), the ongoing debate about gender differences in caregiving for family members remains a relevant topic.
- Measurement scales that assess the process of living with a chronic illness or related daily life factors from a family's perspective were identified by Ambrosio et al. (2020). In Addition, the question about the quality of life of young unpaid carers takes Bou (2023) a closer look, which is essential.

Focus: Chronic illness & family in health literacy research:

- - The family plays an important supportive role in coping with chronic illness. At the same time, it contributes significantly to the acquisition, understanding, and use of health information in decision-making processes.
- In the literature, health literacy and related concepts – such as health information behavior – have long been discussed in connection with the social context (e.g., in the context of surrogate information seeking, resulting conflicts, or the role of the family as an information source).
- A key review identifies the concept of distributed health literacy as particularly promising for examining health literacy in the family context of chronic illness (Muscat et al., 2021).
- According to Edwards et al. (2015), distributed health literacy refers to the abilities, skills, and practices of others that contribute to an individual's level of health literacy.
- Empirical studies on distributed health literacy are still limited. This is especially true for quantitative research, partly due to the lack of existing measurement instruments.
- Investigating distributed health literacy in the context of chronic illness is therefore considered an important research gap.

Focus Chronic illness & family research from a nursing perspective:

- - Importance of telemedicine-led interventions is increasing (Mulyana et al., 2023)
  - Need for the development of family-oriented interventions and research in older cancer patients, investigation of health practices (Konradsen et al., 2020)
  - Overall low number of quantitative research methods
  - Parents of children with a chronic illness are already considered in the literature (Nygård et al., 2018; Ludvigsen et al., 2021)

Focus: Chronic illness & family research in Switzerland:

- Considering the applied methods, all included studies were limited to cross-sectional investigations, mainly applying semi-structured interviews or questionnaires. One study applied an ethnographic approach using participant observation (Dammann 2022), another used the grounded theory approach (study by Gemperli, Brach, & Rubinelli, 2015-2019, e.g., Zanini et al. 2021).
- Overall, only 2 identified studies considered a family specific conceptual framework, namely the Family Adjustment and Adaptation Response (FAAR) Model (in Ris et al. 2020 à McCubbin and Patterson 1983) and the Whole Family Approach (in Frech et al. 2021 à Kaakinen et al., 2015; Chesla, 2010). The use of general frameworks/models was more common including the Convoy Model of Social Relations (in Ris et al. 2022 à Khan Attonicci 1980), Modes of care (in Frech et al. 2021 à Tronto & Fisher, 1990), Coping (in Steck 2007), Self-Management (in Zala 2014), Consolidated Framework for Implementation Research (in Thaqui et al. 2024 and Riguzzi et al. 2024 à Damschroder et al., 2009)
- The ethical considerations made till now are limited to some reflections on informed consent: in the context of intensive care units, in children (in Frech 2021)

**Table S2.1**: Overview of the search strategies for the initial literature searches

| **Focus** | **Inclusion criteria** | **Exclusion criteria** | **Search string** | **Databases** | **Time period** (applied to search) | **Hits** |
| --- | --- | --- | --- | --- | --- | --- |
| Chronic Illness & family caregiving in daily life | Review articles investigating the daily life of families with a chronically ill family member in the English or German language | Articles focusing only on a specific chronic illness, studies exclusively investigating children with chronic illnesses, and intervention studies were excluded. | ("live with" OR "living with" OR "life with" OR "live* experience" OR "living experience" OR "life experience" OR "everyday life" OR "everyday live*" OR "everyday living") AND ("chronic disease*" OR "chronic illness*" OR "chronic condition*" OR "long-term condition*" OR long-term illness*") AND "famil*" AND "caregivers"[MeSH Terms] | PubMed  CINAHL | 2013 - 2023 | Total 54 hits, 12 included. |
| Chronic illness & family in Health Literacy research | Articles related to (dealing with) information connected to chronic illness and family, in English or German language | Articles focused on parental health literacy, were excluded, as were intervention studies and study protocols | **:** "Chronic disease*"[Title/Abstract] OR "chronic illness*"[Title/Abstract] OR "chronic condition*"[Title/Abstract] OR "long-term condition*"[Title/Abstract] OR "long-term Illness*"[Title/Abstract] AND "health literacy"[Title/Abstract] OR "health Information behavior*"[Title/Abstract] OR "health information seeking"[Title/Abstract] AND "Famil*"[Title/Abstract] | PubMed  CINAHL | 2011-2023 | Total 467 hits, included were 10 qualitative studies, 9 quantitative studies, 4 reviews, and 1 book. |
| Chronic illness & family research from a Nursing perspective | Review articles to chronic illness and family from the field of nursing science, in English or German language | Intervention studies and study protocols | (“Chronic disease” [TIAB] OR “Chronic illness” [TIAB] OR “Chronic condition” [TIAB]) AND Famil* [TIAB] AND “Nursing science” | PubMed  CINAHL | 2013 - 2023 | Total 13 Hits, 5 included |
| Chronic illness & family research in Switzerland | Articles or ongoing or completed projects on chronic diseases and families (incl. family caregivers), in a national language or English | Articles that focused on families with a chronically ill child were excluded, as were publications or projects without reference to Switzerland and study protocols | ("caregivers"[MeSH Terms] OR "family caregiver"[Text Word]) AND ("Chronic Disease"[Mesh] OR "Multiple Chronic Conditions"[Mesh]) AND "Humans"[Mesh] AND Switzerland | PubMed, CINAHL, Web of Science, SocIndex, SNSF Project Data-Portal | 2013 - 2023 | Total 69 hits, 7 included articles and identified 5 projects |

**Table S2.2:** Overview of the included studies from the initial literature searches

| **Nr.** | **Authors/Year** | **Title** | **Population/Context** | **Concepts** | **Methods** | **Aim/Results** |
| --- | --- | --- | --- | --- | --- | --- |
| 1 | Abreu et al. 2018 | Distributed health literacy among people living with type 2 diabetes in Portugal: Defining levels of awareness and support | Patients with type 2 diabetes | Distributed health literacy (DHL) | Qualitative study: Semi-structured interviews with 26 patients with type 2 diabetes, grounded theory | The study investigates how social networks and personal experiences paint a picture of health literacy. Explores, among other things, the role of family members as “health literacy mediators” |
| 2 | Adams et al. 2009 | The information needs of partners and family members of cancer patients: a systematic literature review. | Partners and/or family members of adult cancer patients | Information needs | Systematic Review | Thirty-two papers were included in the review. Eleven categories of information need were identified. There was a predominant focus on breast or prostate cancer, leaving a knowledge gap in relation to other cancers. Few papers moved beyond the diagnosis and initial treatment phase, and most did not distinguish between met and unmet needs. Those that did, indicated that partners/family members are more likely to have unmet needs for information about supportive care than for medical information. The concept of ‘information need’ was generally poorly developed and theorised in the papers |
| 3 | Ambrosio et al. 2021 | Living with Chronic Illness from the Family Perspective: An Integrative Review | Measuring the family process of living with a chronic illness from a holistic perspective | Living with a chronic illness (family perspective) | Integrative Review | Identification and analysis of existing scales: process of living with a chronic illness, associated factors of daily life from the family's perspective |
| 4 | Ansari et al. 2021 | Health information-seeking behavior and self-care in women with osteoporosis: a qualitative study | Women with osteoporosis/ Family as information and support system | Health information seeking behavior | Qualitative study: 15 Interviews with content analysis | This study aimed at assessing health information–seeking and self-care behaviors of women with osteoporosis in Iran. They obtained most of their information from other patients, family members, and network of friends |
| 5 | Baik & Willems 2022 | Use of Digital Health Technology for Seeking Health Information Among Older Family Caregivers | Family Caregivers (FCGs). Individuals who are currently caring for or making healthcare decisions for care recipients with a medical or behavioral condition | Health information seeking behavior | Quantitative secondary analysis of the US Health Information National Data | Having a regular healthcare provider is significantly indirectly associated with FCGs’ confidence in obtaining health information via digital health technologies |
| 6 | Bailey et al. 2022 | The Experiences of Informal Carers during the COVID-19 Pandemic: A Qualitative Systematic Review | Experiences of family caregivers of long-term sick people during the pandemic | Pandemic experience / caregiving relatives | Qualitative Systematic Review | Experiences of family caregivers during the COVID 19 pandemic, key issues for a better understanding of the problems and challenges during the crisis |
| 7 | Bangerte et al. 2019 | Health Information-Seeking Behaviors of Family Caregivers: Analysis of the Health Information National Trends Survey | Family Caregivers. Individuals currently caring for or making health care decisions for someone with a medical, behavioral, disability, or other condition | Health information seeking behavior | Quantitative study: HINTS data | Compare health information seeking of caregivers with non-caregivers: Caregivers sought health information for themselves and others using computers, smartphones, or other electronic means more frequently than non-caregivers. Caregivers born outside of the United States reported greater difficulty seeking health information. Nonwhite caregivers, those with less education, with private insurance, and without a regular health care provider had less confidence seeking health information. Caregivers with higher income had more confidence seeking health information. |
| 8 | Bou 2023 | Factors Associated with the Quality-of-Life of Young Unpaid Carers: A Systematic Review of the Evidence from 2003 to 2019 | Identification of factors: Quality of life (QoL) of young people (young carers) who care for chronically ill family members | Quality of life of young carers | Systematic Review | To produce a systematic review of the factors that influence the quality of life of adolescents as defined by the WHO and the tools available to measure quality of life. |
| 9 | Brown et al. 2021 | Information behavior and social control: Toward an understanding of conflictual information behavior in families managing chronic illness | Families managing diabetes or HIV/AIDS, conditions selected to represent variation in chronic disease experiences | Information behaviors  Health related social control | Qualitative study: Interviews with 38 family groups and 97 individuals over 2 years | Study investigated information behaviors in the context of health related social control and the impact of control on patient health behavior: Findings revealed conflictual information behavior, which led to competitions for control and influence between family members and patients. Over time, some patient behaviors changed |
| 10 | Bueno & Chase 2023 | Gender Differences in Adverse Psychosocial Outcomes among Family Caregivers: A Systematic Review | Gender differences: Psychosocial and role-related outcomes for family caregivers of chronically ill older people | Gender differences | Systematic Review | Gender differences in psychological and role related outcomes |
| 11 | Chua et al. 2020 | Caregivers of cancer patients: what are their information-seeking behaviours and resource preferences? | caregivers of cancer patients who were defined as unpaid individuals who might be the parents, children, spouses, relatives or friends, providing one or more activities of daily living and healthcare needs for the cancer patients. | Health information seeking behavior | Quantitative study | This study aims to establish the prevalence of health-information-seeking behaviors among caregivers of cancer patients as a means of ascertaining if their information needs have been met and their information source and resource preference. |
| 12 | Diviani et al. 2020 | Information seeking behavior and perceived health literacy of family caregivers of persons living with a chronic condition. The case of spinal cord injury in Switzerland | Caregivers aged 18+ who assisted with activities of daily living | Health information seeking behavior, Health literacy | Quantitative study | To examine the information seeking behavior and health literacy of caregivers of individuals living with spinal cord injury in Switzerland and their impact on the caregiving experience. Health professionals were the most trusted source of information. Among information-seekers, higher health literacy levels were shown to be associated with lower subjective caregiver burden and, in  turn, with higher caregivers’ satisfaction with own health. |
| 13 | Duggleby et al. 2021 | Systematic review of factors associated with hope in family carers of persons living with chronic illness | Families with an adult family member in need of care | Factors associated with hope | Systematic Review | Identification of factors associated with hope: Quantitative empirical literature among family caregivers of persons living with chronic illness: What factors are significantly associated with carers of persons living with chronic illness? |
| 14 | Edwards et al. 2013 | 'Distributed health literacy': longitudinal qualitative analysis of the roles of health literacy mediators and social networks of people living with a long-term health condition | People with long-term health conditions, living in South Wales | Distributed health literacy | Longitudinal qualitative study:  Serial interviews investigated the development of health literacy to identify changes in attitudes, knowledge and experiences over time | Study aimed to explain the ‘distributed’ nature of health literacy and how people living with a long-term condition draw on their social network for support with health literacy-related tasks. Health literacy was distributed through family and social networks, and participants often drew on the health literacy skills of others to seek, understand and use health information. Those who passed on their health literacy skills acted as health literacy mediators and supported participants in becoming more health literate about their condition. |
| 15 | Eifert et al. 2016 | Emerging Trends in Family Caregiving Using the Life Course Perspective: Preparing Health Educators for an Aging Society | Baby boomers as caregivers for chronically ill family members | Life Course Perspective | Systematic Review and meta-analysis | Life course perspective: characteristics of baby boomers, effects on family caregiving |
| 16 | Fee et al. 2022 | Examining the support needs of older male spousal caregivers of people with a long-term condition: A systematic review of the literature | Older men who care for their chronically ill partner | Needs of older men who care | Systematic Review | Needs of older men who provide care |
| 17 | Fields et al. 2021 | Relationship between adult and family supporter health literacy levels and supporter roles in diabetes management | Adult patients with diabetes and supporter: To identify an involved supporter, patients were asked, “Do you have a family member or friend who gets involved with your healthcare in one of these ways …” | Health literacy (BHS) | Quantitative study: cross-sectional baseline survey data from 239 adult patients with diabetes | Study examined whether supporters provided unique help to adults with diabetes and low HL and whether HL was associated with adults’ perception of supporter helpfulness. Patients who had low health literacy were more likely to have supporters with low health literacy. |
| 18 | Gholami et al. 2014 | Barriers to health information seeking in Iranian patients with cardiovascular disease: a qualitative study | The study included 31 Iranian participants including 16 cardiovascular patients, 5 family members, and 10 health care providers | Health care information seeking/ health information seeking behavior | Qualitative study: Conventional qualitative content analysis | The purpose of this study was to explore obstacles to seeking health care information among cardiovascular patients from the perspectives of patients, their family caregivers, and health care providers. Five themes emerged including ‘poor quality of information provision,’ ‘mutual ambiguity’, ‘beliefs, faith, and expectations,’, ‘from routine life to obtaining information,’ and ‘conditions governing information seekers.’ |
| 19 | Griese 2021 | Gesundheitskompetenz bei chronischer Krankheit in Deutschland: ein Scoping Review [Health literacy in chronic disease in Germany: a scoping review] | Chronic illness | Health literacy | Scoping Review | Included because there is an overview of health literacy research on chronic illness in Germany, but there were no studies dealing with health literacy in the context of the family |
| 20 | Igai 2019 | Concept analysis of dignity-centered care for people with chronic progressive disease. | This concept analysis describes attributes, antecedents, and consequences of dignity-centered care for people with chronic progressive disease. | Rodgers' method of concept analysis was used to clarify the concept's defining attributes. | Concept analysis | Dignity-centered care is defined as: care that results in improvement of self-esteem, deepens purpose and meaning of life, maintains and improves quality of life and provides relief from multi-faceted distress. This happens through protecting the individual's rights, bolstering self-esteem and reducing multi-faceted distress through continued relationships with family, friends, and providers. This concept is a fulcrum for patient and nurse reciprocity enabling both to realize greater integrity and respect, and is useful for nursing practice and research. |
| 21 | Joo & Liu 2022 | The Experience of Chronic Illness Transitional Care: A Qualitative Systematic Review | Chronically ill people and their relatives during the transition from hospital to home. | Transition from hospital to home | Qualitative Systematic Review | The experiences and perceptions of chronically ill people and their relatives during the transition from hospital to the home environment. |
| 22 | Knecht et al. 2015 | The Perspective of Siblings of Children with Chronic Illness A Literature Review | The Review gives an overview about the existing research concerning siblings' perspective within the familial experiences of childhood chronic illness. | The findings were distinguished in a descriptive and content synthesis of the included reviews. | Literature Review | The study identified a map of dimensions of experiences—well-elaborated as well as fragmentary. Many of the included studies were conducted by a proxy and not from the sole siblings' perspective. Further research should concentrate on the sole siblings' perspective, to make siblings' voices audible. |
| 23 | Konradsen et al. 2020 | Health practices in Europe towards families of older patients with cancer: a scoping review | A total of 12 articles were included, showing that family interventions are generally based on end-of-life care. Most studies used a qualitative approach and involved different types of family member as participants. Most studies were conducted in the UK. | Health practice, family interventions | Scoping Review | Review findings revealed limited knowledge about health practices in Europe towards families with an older patient with cancer. This review indicates a need to increase family-focused research that examines health practices that meet the needs of families of older patients with cancer. Seeing cancer as a chronic disease, there is an urgent need for the implementation of family-focused interventions. |
| 24 | Ludvigsen et al. 2021 | Being cross pressured-parents’ experiences of the transfer from paediatric to adult care services for their young people with long term conditions: A systematic review and qualitative research synthesis | Family members of young people (13–24 years) with long-term conditions tend to experience multiple challenges when their children transfer from paediatric to adult care, as do the patients themselves. | Transition management pediatric care to adult care | Qualitative research synthesis | The clinical implications are to recognize parents’ experiences and distress in healthcare planning to promote safe and predicable transfers of their young people. Provision of healthcare to parents during transfer needs to be tailored to a collaborative decision- making process between parents, their young people, and involved practitioners across pediatric and adult healthcare services. |
| 25 | Marin-Maicas et al. 2021 | Living with Long Term Conditions from the Perspective of Family Caregivers. A Scoping Review and Narrative Synthesis | Family with an adult in need of care | Living with a chronic illness (family perspective) | Scoping Review & Narrative Synthesis | This study aimed to understand life with a chronic illness from the family's perspective |
| 26 | Merati-Fashi et al. 2022 | Stroke Survivors and Their Family Caregivers' Experiences of Health Information Seeking: A Qualitative Study | Participants included five stroke survivors and fifteen family caregivers | Health information seeking behavior | Semi-structured  interviews and qualitative content analysis | This study aimed to explore the experiences of health information seeking by stroke survivors and their family caregivers |
| 27 | Mulyana et al. 2023 | The Efficacy of Internet-Based Interventions in Family-Centered Empowerment Among Children with Chronic Diseases: A Mixed-Methods Systematic Review | This study aimed to determine the efficacy of internet-based family empowerment interventions among children with chronic diseases. | Family empowerment (FE) is one approach to strengthen family care among children with chronic diseases | Systematic review | This study analyzed six study and identified four themes regarding efficacy of internet-based interventions in empowering families with children with chronic diseases: knowledge; availability of resources and timesaving; connectedness and communication; and family management and self-confidence. |
| 28 | Murray et al. 2022 | Distributive health literacy and medication management: A longitudinal qualitative study with Bhutanese former refugees in Tasmania | Bhutanese former refugees and their carers | Distributed health literacy | Longitudinal qualitative study  15 former refugees, and their carers were interviewed 3-4 times over 9 months | Tasmania, Australia is home to a population of Bhutanese former refugees who have resettled since 2007. This research explored how this community perceives chronic conditions and managed their medication using the concept of distributed health literacy. Four themes related to distributed health literacy were identified (see Murray et al. 2022) |
| 29 | Nygård & Clancy  (2018) | Unsung heroes, flying blind—A Metasynthesis of parents’ experiences of caring for children with special health-care needs at home | To aggregate, synthesize and interpret qualitative research studies of parents’ experiences of caring for a child with special health-care needs at home | Special healthcare needs | Metasynthesis | The enormous burden of care can weaken the parents’ will to carry on and result in a decreased ability to provide care. This can have an impact on the parents’ health, family functioning and the sick child's potential health outcomes. Nurses are in a unique position to help these families and should be better prepared for the role. |
| 30 | Oh 2015 | Predictors of Self and Surrogate Online Health Information Seeking in Family Caregivers to Cancer Survivors | 1,113 family caregivers were included in this study | Surrogate Online Health Information Seeking | Quantitative Study | The purpose of this research is to investigate various factors predicting online health information seeking for themselves (self OHIS) and online health information seeking for others (surrogate OHIS) in family caregivers to cancer survivors. Caregivers' self and surrogate OHIS were commonly predicted by their self-rated health and attention to the Internet. However, age, race, and education were significantly associated with self OHIS only, while gender and marital status were significantly associated with surrogate OHIS only. |
| 31 | Park & Schumacher 2014 | The state of the science of family caregiver-care receiver mutuality: a systematic review | Families with an adult in need of care | Care relationship, relationship theory | Systematic Review | Development of a theoretical framework based on relationship theory |
| 32 | Rathnayake et al. 2019 | Family carers' perspectives of managing activities of daily living and use of mHealth applications in dementia care: A qualitative study | The family carers were unpaid primary carers  who were aged 18 years and older and provided informal care at home for a person diagnosed with dementia. | Health information seeking behavior  Information needs | Qualitative study: In‐depth interviews with a purposive sample of family  Inductive thematic analysis | To examine the needs, barriers and challenges experienced by family carers of people with dementia concerning the management of their care recipients' functional disabilities, and their experiences and opinions of using mobile health (mHealth) applications in health information seeking. |
| 33 | Reifegerste et al. 2017 | Surrogate health information seeking in Europe: Influence of source type and social network variables | Surrogate seeking by “health-onliners” and “health-offliners” across the European Union (not only limited to families) | Surrogate health information seeking | Quantitative study | The goal of this study was to investigate the characteristics of surrogate health information seekers using online and offline sources and the recipients of surrogate information. |
| 34 | Reifegerste et al. 2020 | Understanding Information Seeking about the Health of Others: Applying the Comprehensive Model of Information Seeking to Proxy Online Health Information Seeking | Proxy seekers, i.e., individuals who seek information about the health of others | Surrogate health information seeking | Quantitative study: Structural equation modeling served to evaluate the associations between the health-related factors and proxy health information seeking intentions, as well as support intentions. | The comprehensive model of information seeking (CMIS) is an established model that predicts information seeking for the individual seeker. The model was modified and extended with concepts of social network ties to predict proxy information. seeking intentions and the resulting social support intentions. |
| 35 | Rosland et al. 2010 | Family Influences on Self-Management Among Functionally Independent Adults with Diabetes or Heart Failure: Do Family Members Hinder As Much As They Help? | 439 patients with diabetes or heart failure: Respondents were asked how often they receive support from family members or friends in each of five self-management domains: u.a.” information/decisions.” | Support for Self-Management from Family, including Information and Decisions | Quantitative study: Cross-sectional survey | Study examined family member support and family-related barriers to self-care. Patient characteristics associated with family support and family barriers and how each was associated with self-management adherence were identified. |
| 36 | Stratakis et al. 2021 | Newer Guidelines for Caregivers of Patients with Dementia: A Systematic Review | The purpose of this systematic review was to identify the most recent information available regarding guidance to the family and caregivers of dementia patients | Family counseling | Systematic review | One of the major challenges facing community nursing is using scientific evidence effectively to improve the quality of life for caregivers and the care they provide to patients with dementia. Community nurses must regularly update and share scientific evidence and available resources to improve caregivers' quality of life, which will, in turn, improve the care they provide. |
| 37 | Suutari et al. 2023 | Improving heart failure care with an Experience-Based Co-Design approach: what matters to persons with heart failure and their family members? | 17 people with heart failure and four family members participated in this single case study | Difficulties in understanding and applying information about heart failure and its care | In line with experienced-based co-design methodology, field notes from observations of healthcare consultations, individual interviews and meeting minutes from stakeholders’ feedback events | The Aim of this study was to use experience-based co-design to identify experiences of heart failure and its care in a Swedish cardiac care setting, and to understand how these experiences can translate into improvements in heart failure care for patients and their families. |
| 38 | Synnot et al. 2016 | Online health information seeking and how people with multiple sclerosis find, assess, and integrate treatment information to manage their health. | Sixty participants (51 people with MS; 9 family members) | Information seeking | Qualitative study: focus group or online forum. Thematic analysis underpinned by a coding framework | The study aimed to explore the needs and experiences of people with MS regarding the integration of treatment information into decision-making processes when searching online. Participants were asked to describe how they find and assess reliable treatment information. |
| 39 | Vick & Wolff 2021 | A scoping review of person and family engagement in the context of multiple chronic conditions | Families with an adult in need of care | Engagement of Families | Scoping Review | Reviewing conceptual models of engagement in order to identify key concepts. |
| 40 | Washington et al. 2011 | Information needs of informal caregivers of older adults with chronic health conditions | Informal caregivers of older adults with chronic health  conditions | Information needs | Review: Structured search in databases to identify studies of caregiver information needs, followed by data extraction and syntheses. | To systematically examine current evidence pertaining to information needs of informal caregivers of older adults with chronic health conditions. The 62 articles that met the stated inclusion criteria highlighted extensive needs among informal caregivers for practical, accessible, timely information. |
| 41 | Whitehead et al. 2018 | The role of the family in supporting the self-management of chronic conditions: A qualitative systematic review | Family with an adult in need of care | Support by family members | Qualitative Systematic Review | Contribution of family members to the promotion and support of self-management |
| 42 | Wittenberg et al. 2021 | Caring for the family caregiver. Palliative care communication and health literacy. | Family caregiver in chronic ill-ness | Health literacy  Communication | Book | Caring for the Family Caregiver examines the high cost and poorly addressed exigencies of the family caregiver in chronic illness, including health literacy, palliative care, and health outcomes, through the prism of communication. Using an interdisciplinary approach, this book identifies the impact of communication and its burdens on caregivers. It presents four caregiver profiles—the Manager, the Carrier, the Partner, and the Lone Caregiver—each emerging from a family system with different patterns of conversational sharing and expectations of conformity. This essential resource is ideal for providers, students, clinicians, policymakers, and family caregivers. |
| 43 | Yuen et al. 2018 | Health literacy of caregivers of adult care recipients: A systematic scoping review | Caregivers of adult care recipients | Health literacy | Scoping Review: Electronic databases were searched for relevant English-language publications that assessed health literacy in caregivers. | The aim of the study was to systematically review literature related to health literacy of caregivers of adult care recipients, and examine its relationship with care recipient, and caregiver, health outcomes |
| 44 | Binder et al. 2014 | Hilfreiche und belastende Faktoren im Umgang mit COPD bei Patienten und ihren Partnern – eine qualitative Studie [Barriers and facilitating factors in order to cope with COPD for patients and partners – a qualitative study] | Patient with COPD and partner | Barriers and facilitating factors | Qualitative study | The aim of the study is to identify barriers of facilitating factors in dealing with COPD for patients and their partner. |
| 45 | Dammann et al. 2022 | Insights into the challenges faces by chronically critically ill patients, their families and healthcare providers: An interpretive description | Patients with chronic critical illness, their families, and healthcare providers | Challenges of patients, family members, and healthcare providers during disease trajectory | Qualitative study: Thone´s methodology, comprising 5 patients, 12 family members, and 92 healthcare providers. | To describe and understand the challenges of patients with chronic critical illness, their families and healthcare providers during the disease trajectory. |
| 46 | Frech 2021 | Defining Support for Young Carers | Children, adolescents, and young adults who take care of an ill family members | Model of support | Mixed methods study: Concept analysis, quantitative questionnaire (n=3,518), qualitative semi-structured interviews (n=40) | A model of support for young carers was developed. |
| 47 | Fusi-Schmidhauser et al. 2020 | Living with advanced chronic obstructive pulmonary disease: a qualitative interview study with patients and informal carers | Patients with COPD and their informal carers | Support by family members | Qualitative study | Exploring patients´ and informal carers´ experiences in living with advanced COPD |
| 48 | Ris et al. 2019 | Psychometric evaluation of the German version of a social support scale of FAFHES (Family Functioning, Family Health and Social Support) | Family members of older adults who receive services from a home health nursing organization | Care and support by family members | Quantitative study questionnaire (n=207) | Translate the 20-item FAFHES questionnaire from English to German and test validity and reliability |
| 49 | Ris et al. 2020 | Exploring factors associated with family caregivers´preparedness to care for an older family member together with home care nurses: an analysis in swiss urban area | Family members of older adults who receive services from a home health nursing organization | Preparedness to care for family member | Quantitative study: questionnaire (n=243) | Exploring factors associated with family caregivivers´preparedness to care for an older family member. |
| 50 | Zimmermann-Acklin 2011 | Chronische Krankheiten: Unzeitgemässe Beobachtungen zu einem Zeichen der Zeit [Chronic diseases: Untimely observations on a sign of the times] | Families with chronically ill children | Care and support by family members | Commentary/ Editorial | Changes in society and changes in dealing with illness and care. |

**References from the initial literature searches**

Abreu, L., Nunes, J. A., Taylor, P. & Silva, S. (2018). Distributed health literacy among people living with type 2 diabetes in Portugal: Defining levels of awareness and support. *Health & Social Care in the Community*, *26*(1), 90–101. <https://doi.org/10.1111/hsc.12465>

Adams, E., Boulton, M. & Watson, E. (2009). The information needs of partners and family members of cancer patients: a systematic literature review. *Patient Education and Counseling*, 77(2), 179–186. <https://doi.org/10.1016/j.pec.2009.03.027>

Ambrosio, L., Navarta-Sánchez, M. V., Carvajal, A., & Garcia-Vivar, C. (2021). Living with Chronic Illness from the Family Perspective: An Integrative Review. *Clinical Nursing Research, 30*(5), 579-590. <https://doi.org/10.1177/1054773820947983>

Ansari, A., Fahimfar, N., Noruzi, A., Fahimifar, S., Hajivalizadeh, F., Ostovar, A., Larijani, B. & Sanjari, M. (2021). Health information-seeking behavior and self-care in women with osteoporosis: a qualitative study. *Archives of Osteoporosis*, *16*(1), 78. <https://doi.org/10.1007/s11657-021-00923-8>

Baik, D. & Willems, E. (2022). Use of Digital Health Technology for Seeking Health Information Among Older Family Caregivers. *Studies in Health Technology and Informatics*, *290*, 997–999. <https://doi.org/10.3233/SHTI220236>

Bailey, C., Guo, P., MacArtney, J., Finucane, A., Swan, S., Meade, R., & Wagstaff, E. (2022). The Experiences of Informal Carers during the COVID-19 Pandemic: A Qualitative Systematic Review. *International Journal Environmental Research Public Health, 19*(20). <https://doi.org/10.3390/ijerph192013455>

Bangerter, L. R., Griffin, J., Harden, K. & Rutten, L. J. (2019). Health Information-Seeking Behaviors of Family Caregivers: Analysis of the Health Information National Trends Survey. *JMIR Aging*, *2*(1), e11237. <https://doi.org/10.2196/11237>

Binder, H., Mörgeli, H., Meier, C., Witzemann, L., Drabe, N., Jenewein, J. (2014). Hilfreiche und belastende Faktoren im Umgang mit COPD bei Patienten und ihren Partnern – eine qualitative Studie. *Praxis*, 103(2): 75-83.

Bou, C. (2023). Factors Associated with the Quality-of-Life of Young Unpaid Carers: A Systematic Review of the Evidence from 2003 to 2019. *International Journal of Environmental Research Public Health, 20*(6). <https://doi.org/10.3390/ijerph20064807>

Brown, L. K. & Veinot, T. C. (2021). Information behavior and social control: Toward an understanding of conflictual information behavior in families managing chronic illness. *Journal of the Association for Information Science & Technology*, *72*(1), 66–82. <https://doi.org/10.1002/asi.24362>

Bueno, M. V., & Chase, J. D. (2023). Gender Differences in Adverse Psychosocial Outcomes among Family Caregivers: A Systematic Review. *Western Journal of Nursing Research, 45*(1), 78-92. <https://doi.org/10.1177/01939459221099672>

Chua, G. P., Ng, Q. S., Tan, H. K. & Ong, W. S. (2020). Caregivers of cancer patients: what are their information-seeking behaviours and resource preferences? *Ecance, 14, 1068.* <https://doi.org/10.3332/ecancer.2020.1068>

Dammann, M. Staudacher, S., Simon, M., Jeitziner, M. (2022). Insights into the challenges faces by chronically critically ill patients, their families and healthcare providers: An interpretive description. *Intensive & Critical Care Nursing*, 68, 103135 <https://doi.org/10.1016/j.iccn.2021.103135>

Diviani, N., Zanini, C., Jaks, R., Brach, M., Gemperli, A. & Rubinelli, S. (2020). Information seeking behavior and perceived health literacy of family caregivers of persons living with a chronic condition. The case of spinal cord injury in Switzerland. *Patient Education and Counseling*, *103*(8), 1531–1537. <https://doi.org/10.1016/j.pec.2020.02.024>

Duggleby, W., HeunJung, L., Nekolaichuk, C., & Fitzpatrick-Lewis, D. (2021). Systematic review of factors associated with hope in family carers of persons living with chronic illness. *Journal of Advanced Nursing, 77*(8), 3343-3360. <https://doi.org/10.1111/jan.14858>

Edwards, M., Wood, F., Davies, M. & Edwards, A. (2015). 'Distributed health literacy': longitudinal qualitative analysis of the roles of health literacy mediators and social networks of people living with a long-term health condition. *Health expectations*, *18*(5), 1180–1193. <https://doi.org/10.1111/hex.12093>

Eifert, E. K., Adams, R., Morrison, S., & Strack, R. (2016). Emerging Trends in Family Caregiving Using the Life Course Perspective: Preparing Health Educators for an Aging Society. *American Journal of Health Education, 47*(3), 176-197. <https://doi.org/10.1080/19325037.2016.1158674>

Fee, A., McIlfatrick, S., & Ryan, A. (2020). Examining the support needs of older male spousal caregivers of people with a long-term condition: A systematic review of the literature. *International Journal Older People Nursing, 15*(3), e12318. <https://doi.org/10.1111/opn.12318>

Fields, B., Lee, A., Piette, J. D., Trivedi, R., Mor, M. K., Obrosky, D. S., Heisler, M. & Rosland, A.‑M. (2021). Relationship between adult and family supporter health literacy levels and supporter roles in diabetes management. *Families, Systems & Health*, *39*(2), 224–233. <https://doi.org/10.1037/fsh0000503>

Frech, M. (2021). Defining support for Young Carers in Switzerland. Doctoral Thesis University of Vienna, Department of Nursing Sciences.

Fusi-Schmidhauser, T., Froggott, K., Preston, N. (2020). Loving with Advanced Chronic Obstructuve Pulmonary Disease: a qualitative interview study with patients and informal carers. *Journal of Chronic Obstructive Pulmonary Disease.* 17(4) 410-418, <https://doi.org/10.1080/15412555.2020.1782867>

Gholami, M., Fallahi Khoshknab, M., Maddah, S. S. B., Ahmadi, F. & Khankeh, H. (2014). Barriers to health information seeking in Iranian patients with cardiovascular disease: a qualitative study. *Heart & Lung*, *43*(3), 183–191. <https://doi.org/10.1016/j.hrtlng.2014.01.010>

Griese, L. (2021). Gesundheitskompetenz bei chronischer Krankheit in Deutschland: ein Scoping Review. *Prävention und Gesundheitsförderung*, *17*(1), 104–112. <https://doi.org/10.1007/s11553-021-00843-y>

Joo, J. Y., & Liu, M. F. (2022). The Experience of Chronic Illness Transitional Care: A Qualitative Systematic Review. *Clinical Nursing Research, 31*(2), 163-173. <https://doi:10.1177/10547738211056166>

Knecht, C., Hellmers, C., & Metzing, S. (2015). The perspective of siblings of children with chronic illness: a literature review. *Journal of Pediatric Nursing*, *30*(1), 102–116. <https://doi.org/10.1016/j.pedn.2014.10.010>

Konradsen, H., Brødsgaard, A., Østergaard, B., Svavarsdóttir, E., Dieperink, K. B., Imhof, L., Luttik, M. L., Mahrer-Imhof, R., & García-Vivar, C. (2020). Health practices in Europe towards families of older patients with cancer: a scoping review. *Scandinavian Journal of Caring Sciences*, *35*(2), 375–389. <https://doi.org/10.1111/scs.12855>

Ludvigsen, M. S., Hall, E. O. C., Westergren, T., Aagaard, H., Uhrenfeldt, L., & Fegran, L. (2021). Being cross pressured-parents' experiences of the transfer from pediatric to adult care services for their young people with long term conditions: A systematic review and qualitative research synthesis. *International journal of Nursing Studies*, *115*, 103851. <https://doi.org/10.1016/j.ijnurstu.2020.103851>

Marco, D. J., Thomas, K., Ivynian, S., Wilding, H., Parker, D., Tieman, J., & Hudson, P. (2022). Family carer needs in advanced disease: systematic review of reviews. *BMJ Support Palliat Care, 12*(2), 132-141. doi:10.1136/bmjspcare-2021-003299

Marín-Maicas, P., Corchón, S., Ambrosio, L., & Portillo, M. C. (2021). Living with Long Term Conditions from the Perspective of Family Caregivers. A Scoping Review and Narrative Synthesis. *International Journal of Environmental Research and Public Health, 18*(14). <https://doi.org/10.3390/ijerph18147294>

Merati-Fashi, F., Dalvandi, A. & Parsa Yekta, Z. (2022). Stroke Survivors and Their Family Caregivers' Experiences of Health Information Seeking: A Qualitative Study. *International Journal of Community Based Nursing and Midwifery*, *10*(4), 269–278. <https://doi.org/10.30476/IJCBNM.2022.94489.1997>

Mulyana, A. M., Rakhmawati, W., Wartakusumah, R., Fitri, S. Y. R., & Juniarti, N. (2023). The Efficacy of Internet-Based Interventions in Family-Centered Empowerment Among Children with Chronic Diseases: A Mixed-Methods Systematic Review. *Journal of Multidisciplinary Healthcare*, *16*, 3415–3433. <https://doi.org/10.2147/JMDH.S440082>

Murray, L., Elmer, S., Breen, J. & Nash, R. (2022). Distributive health literacy and medication management: A longitudinal qualitative study with Bhutanese former refugees in Tasmania. *Health Promotion Journal of Australia*, *33*(2), 403–411. <https://doi.org/10.1002/hpja.501>

Muscat DM, Gessler D, Ayre J, Norgaard O, Heuck IR, Haar S, Maindal HT. Seeking a deeper understanding of 'distributed health literacy': A Systematic Review. *Health Expections,* 2022 Jun;25(3):856-868. <https://doi.org/10.1111/hex.13450>

Nygård, C., & Clancy, A. (2018). Unsung heroes, flying blind—A metasynthesis of parents’ experiences of caring for children with special health‐care needs at home. *Journal of Clinical Nursing*, 27(15–16), 3179–3196. <https://doi.org/10.1111/jocn.14512>

Oh, Y. S. (2015). Predictors of Self and Surrogate Online Health Information Seeking in Family Caregivers to Cancer Survivors. *Social Work in Health Care,* 54(10), 939–953. <https://doi.org/10.1080/00981389.2015.1070780>

Park, E. O., & Schumacher, K. L. (2014). The state of the science of family caregiver-care receiver mutuality: a systematic review. *Nursing Inquire*, 21(2), 140-152. <https://doi.org/10.1111/nin.12032>

Rathnayake, S., Jones, C., Calleja, P. & Moyle, W. (2019). Family carers' perspectives of managing activities of daily living and use of mHealth applications in dementia care: A qualitative study. *Journal of Clinical Nursing*, 28(23-24), 4460–4470. <https://doi.org/10.1111/jocn.15030>

Reifegerste, D., Bachl, M. & Baumann, E. (2017). Surrogate health information seeking in Europe: Influence of source type and social network variables. *International Journal of Medical Informatics*, 103, 7–14. <https://doi.org/10.1016/j.ijmedinf.2017.04.006>

Reifegerste, D., Blech, S. & Dechant, P. (2020). Understanding Information Seeking about the Health of Others: Applying the Comprehensive Model of Information Seeking to Proxy Online Health Information Seeking. *Journal of Health Communication*, 25(2), 126–135. <https://doi.org/10.1080/10810730.2020.1716280>

Reifegerste, D., Blech, S. & Dechant, P. (2020). Understanding Information Seeking about the Health of Others: Applying the Comprehensive Model of Information Seeking to Proxy Online Health Information Seeking. Journal of Health Communication, 25(2), 126–135. <https://doi.org/10.1080/10810730.2020.1716280>

Ris, I., Schnepp, W., Mahrer-Imhof, R. (2019) Psychometric evaluation of the German version of a social support scale of FAFHES (Family Functioning, Family Health and Social Support). Scandinavian Journal of Caring Science, <https://doi.org/10.1111/scs.12700>

Ris, I., Volken, T., Schnepp, W., Mahrer-Imhof, R. (2022) Exploring factors associated with family caregivivers´preparedness to care for an older family member together with home care nurses: an analysis in swiss urban area. *Journal of Primary Care & Community Health*, 13:1-9. <https://doi.org/10.1177/21501319221103961>

Rosland, A.‑M., Heisler, M., Choi, H.‑J., Silveira, M. J. & Piette, J. D. (2010). Family influences on self-management among functionally independent adults with diabetes or heart failure: do family members hinder as much as they help? *Chronic illness*, 6(1), 22–33. <https://doi.org/10.1177/1742395309354608>

Stratakis, E., Mantzorou, M., Kalemikerakis, I., Kavga, A., & Plakas, S. (2021). Newer Guidelines for Caregivers of Patients with Dementia: A Systematic Review. *Perioperative Nursing (GORNA)*, 9(4), 233–245. <https://doi.org/10.5281/zenodo.4486379>

Suutari, A.‑M., Thor, J., Nordin, A. & Josefsson, K. A. (2023). Improving heart failure care with an Experience-Based Co-Design approach: what matters to persons with heart failure and their family members? *BMC Health Services Research*, 23(1), 294. <https://doi.org/10.1186/s12913-023-09306-w>

Synnot, A. J., Hill, S. J., Garner, K. A., Summers, M. P., Filippini, G., Osborne, R. H., Shapland, S. D. P., Colombo, C. & Mosconi, P. (2016). Online health information seeking: how people with multiple sclerosis find, assess and integrate treatment information to manage their health. *Health Expectations*, 19(3), 727–737. <https://doi.org/10.1111/hex.12253>

Vick, J. B., & Wolff, J. L. (2021). A scoping review of person and family engagement in the context of multiple chronic conditions. *Health Services Research*, 56(1), 990-1005. <https://doi.org/10.1111/1475-6773.13857>

Washington, K. T., Meadows, S. E., Elliott, S. G. & Koopman, R. J. (2011). Information needs of informal caregivers of older adults with chronic health conditions. Patient *Education and Counseling*, 83(1), 37–44. <https://doi.org/10.1016/j.pec.2010.04.017>

Whitehead, L., Jacob, E., Towell, A., Abu-Qamar, M., & Cole-Heath, A. (2018). The role of the family in supporting the self-management of chronic conditions: A qualitative systematic review. Journal of Clinical Nursing, 27(1-2), 22-30. <https://doi.org/10.1111/jocn.13775>

Wittenberg, E., Goldsmith, J. V., Ragan, S. L. & Parnell, T. A. (2021). Caring for the family caregiver: Palliative care communication and health literacy. Oxford University Press.

Yuen, E. Y. N., Knight, T., Ricciardelli, L. A. & Burney, S. (2018). Health literacy of caregivers of adult care recipients: A systematic scoping review. Health & Social Care in the Community, 26(2), e191-e206. <https://doi.org/10.1111/hsc.12368>

Zimmermann-Acklin, M. (2011). Chronische Krankheiten: Unzeitgemässe Beobachtungen zu einem Zeichen der Zeit. *Schweizerische Ärztezeitung*, 92(7), 247–249

**Table S3: Overview of instruments to measure family well-being**

The instruments below were identified through a two-step process: First, from the literature gathered during our initial searches (see S 2), and subsequently through additional targeted searches to ensure comprehensive coverage. Each instrument is summarized, including its theoretical background.

| **Concept** | **Tool** | **Dimensions** | **Item(s)** | **Languages** | **Population** | **Who answers** | **Goal** | **Validation Process** | **Theoretical background** | **Year** | **Reference** |
| --- | --- | --- | --- | --- | --- | --- | --- | --- | --- | --- | --- |
| Family Functioning | Family Environment Scale (FES) | 1. Family Relationship,  2. Personal Growth,  3. System Maintenance and Change | 90 | >22 languages, incl. Eng, Chi, Jap, Mal, Spa, Por, Gre, Heb | Family general | Multiple family members, designed for use with children 11 years and older. | Measurement of social climate | The initial choice and wording of FES items were guided by information obtained from observations and interviews with families. Initial items were distributed to 1000 individuals from 285 families. | It focuses on the measurement and description of the interpersonal relationships among family members, on the directions of personal growth which are emphasized in the family, and on the basic organizational structure of the family. | 1974 | Moos, R. H. (1974). Family environment scale preliminary manual. Palo Alto, CA: Consulting |
|  | The Family AGPAR* Index: Adaptation, Partnership, Growth, Affection, and Resolve | 1. Adaptation,  2. Partnership,  3. Growth,  4. Affection,  5. Resolve | 5 | Eng, Chi, Jap, Spa | Family with chronic illness | Generally, one of the parents fills it in, ok to use with children from 10 years onwards. | Screening for physicians to identify family difficulties could also be used for research. | Compared instrument´s index score to scores of the family function index (FFI) and to findings of clinical therapists after, on average, 5 sessions and a minimum of one hour. The therapists rated their patients´ families on a 10-point scale. Some of the participants were nonclinical (n=38) and the others were psychiatric outpatients (n=20) | Unknown | 1978 | Smilkstein G. (1978). The family APGAR: a proposal for a family function test and its use by physicians. *The Journal of family practice, 6*(6), 1231–1239. |
|  | McMaster Family Assessment Device (FAD) | 1. Problem Solving,  2. Communication,  3. Affective Responsiveness,  4. Affective Involvement,  5. Behavior Control,  6. General Functioning | 60 | Eng, Ger, Fra, Chi, Gre, Hin, Ice, Ita, Por, Spa, Swe, Tur | Family general and Family with (chronic) (mental) illness (parent or child) | Adult or child from 12 years onwards, generally used with just one family member | Screening & Research - developed with the goal of providing a treatment program for family therapists~~,~~ and a theoretical framework for research | 294 individuals from 112 families were involved in the development; they came from different clinical settings (e.g. psychiatric hospital, stroke rehab), plus 209 students from a psychology course | McMaster Model of Family Functioning (MMFF) | 1983 (updated 1990) | Epstein, N. B., Baldwin, L. M., Bishop, D. S. (1983). The McMaster family assessment device. *Journal of Marital and Family Therapy, 9*(2), 171-180. <https://doi.org/10.1111/j.1752-0606.1983.tb01497.x> |
|  | Family Relationship Index (FRI) | 1. Family cohesion,  2. Family expressiveness,  3. Family conflict | 27 | (see FES) | Family with chronic illness | Multiple members of one family could participate | Provides an overall index of the quality of the family environment | 53 families, four couples without children, 35 with, and 14 single parents, compared answers with structured interview findings by family therapists | Based on 3~~.~~ subscales of the FES (Family cohesion, Family expressiveness, Family conflict) | 1989 | Hoge, R. D., Andrews, D. A., Faulkner, P., & Robinson, D. (1989). The Family Relationship Index: validity data. *Journal of clinical psychology, 45*(6), 897–903. [https://doi.org/10.1002/1097-4679(198911)45:6<897::AID-JCLP2270450611>3.0.CO;2-T](https://doi.org/10.1002/1097-4679(198911)45:6%3c897::AID-JCLP2270450611%3e3.0.CO;2-T) |
|  | Assessment of Strategies in Families-Effectiveness (ASF-E) | 1. System maintenance  2. System change  3. Coherence  4. Individuailty | 18 | Eng, Ger | Family general | One person of the family, unclear if this could be a child. | Screening - assesses the effectiveness of family functioning or family health | Litrature review, personal experiences, discussions with family clinicians were used for initial selection of items. Afterwards, a group of 14 clinicians, nurses, social workers and clnical psycholgists made a selection. In the second step, 5 family therapists rated the statements. | Friedemann Model [Theorie des systemischen Gleichgewichts] | 1991 | Friedemann, M.-L. (1996). Familien- und umweltbezogene Pflege : die Theorie des systemischen Gleichgewichts. Huber. |
|  | Family Functioning Family Health and Social Support (FAFHES) | 1. Affect,  2. Affirmation,  3. Concrete aid | 20 (Eng), 19 (Ger) | Eng, Ger, Per | Families affected by chronic illness | One family member (caregiver) |  | Initial items were developed based on knowledge generated by three Finnish academic dissertations concerning family functioning, health, and social support provided by nurses. Data were collected from family members of 161 heart patients. | Convoy model of social relations; Maijala’s (2004) substantive interaction theory | 2002 | Åstedt-Kurki, P., Tarkka, M.-T., Paavilainen, E., & Lehti, K. (2002). Development and Testing of a Family Nursing Scale. *Western Journal of Nursing Research, 24*(5), 567–579. <https://doi.org/10.1177/019394590202400508> |
|  | Brief Assessment of Family Functioning Scale (BAFFS) | 1. General Functioning | 20 | (see FAD) | Family or partnership, with or without children | Filled in by caregiver, for couples with and without children, living in same household |  | Data from 1666 families of 3864 individuals, all of whom participated in studies using the complete FAD, were used. | McMaster Model of Family Functioning (MMFF) | 2019 | Mansfield, A. K., Keitner, G. I., & Sheeran, T. (2019). The Brief Assessment of Family Functioning Scale (BAFFS): a three-item version of the General Functioning Scale of the Family Assessment Device. *Psychotherapy Research, 29*(6), 824–831. <https://doi.org/10.1080/10503307.2017.1422213> |
| Family Quality of Life | Beach Family Quality of Life Scale (FQOL) | 1. Family Interaction,  2. Parenting,  3. Emotional Well-being,  4. Physical Well-being / Material Well-being,  5. Disability-Related Support. | 25 | Eng, Fre, Spa | For families with children with or without disability, also possible for a parent with a disability | One adult family member | This scale is  designed to be used as a research tool (e.g. as a pre- and post-test to measure the effectiveness of an intervention, as an outcome measure for programs or services, as a measure of a dependent or independent variable) | Administered to a sample of 1197 individuals from 459 families. Exploratory factor analysis produced a 5-factor solution. Further validation of the factor structure of the FQOL Scale occurred in two rounds of  study involving a total of 488 families. The final FQOL Scale was refined through confirmatory factor analyses into 25 items that assess 5 domains. | Extensive literature reviews, focus groups, and individual interviews with family members of children with disabilities, individuals with disabilities, service providers, and administrators of service agencies | 2003 | Park, J., Hoffman, L., Marquis, J., Turnbull, A. P., Poston, D., Mannan, H., Wang, M., & Nelson, L. L. (2003). Toward assessing family outcomes of service delivery: validation of a family quality of life survey. *Journal of Intellectual Disability Research*, *47*(4–5), 367–384. <https://doi.org/10.1046/j.1365-2788.2003.00497.x> |
|  | (Canadian) Family Quality of Life survey | 1. Health of the Family,   2. Financial Well-Being,  3. Family Relationships,  4. Support from Other People,  5. Support from Disability Related Services,  6. Influence of Values,  7. Careers and Preparing for Careers,  8. Leisure and Recreation,  9. Community Involvement | 54 | Eng (Can, USA Aus), Ger (Austrian), Bos, Chi, Dut, Fla, Far, Fre, Ita, Jap, Mal, Pol, Por, Slo, Spa, Tel | For families with children with intellectual and/or developmental disabilities | Main adult caregiver | The FQOL Survey is intended for two uses. First, it may be useful for service practitioners and family members as part of an overall assessment of support needs and program design. Second, it may be used as an instrument to describe and measure family quality of life, within the limitations of its conceptualization, for research or evaluation purposes. | First pilot with 34 families who were interviewed in 2003, who had a child with intellectual disability, this child was between 10-34 years old. | It was based on Schalock's (2004) study of the quality of life in people with disabilities. (Schalock, R. L. (2004a). (Moving From Individual To Family Quality Of Life As A Research Topic (Chapter 2). In A. Turnbull, I. Brown, H. R. Turnbull & D. L. Braddock (Eds.), Families And People With Mental Retardation And Quality Of Life: International Perspectives (pp. 11 – 24). Washington: American Association on Mental Retardation) | 2006 | Brown I., Brown R. I., Baum N. T., Isaacs B. J., Myerscough T., Neikrug S., Roth D., Shearer J. & Wang M. (2006). Family Quality of Life Survey: Main caregivers of people with intellectual or developmental disabilities. Toronto: Surrey Place Centre. |
| Other | Family Reported Outcome Measure (FROM-16) | 1. Emotional,  2. Personal and Social Life | 16 | >30 translations (incl. Eng, Ger) | Family with (chronic) illness, the person with the illness can be any age infant to elderly | One person, partner or other adult family member | Qunatify disease burden, identify areas where family members need further support, as well as identify those individuals most affected by the patient’s illness. | It was developed based on information from partners and family members of patients from 26 different medical specialties. | A total of 30 items were generated from the content of previous interviews with family members. Qualitative and quantitative feedback from expert panels was collected. | 2014 | Golics, C. J., Basra, M. K. A., Finlay, A. Y., & Salek, S. (2014). The development and validation of the Family Reported Outcome Measure (FROM-16)©to assess the impact of disease on the partner or family member. *Quality of Life Research, 23*(1), 317–326. <https://doi.org/10.1007/s11136-013-0457-y> |
|  | Family Integration Experience Scale:Chronic Illness (FIES:CI) | 1. Negative Integration,  2. Positive Integration | 9 | Eng, Por (Brasil) | Family in the context of chronic condition or chronic illness | The person with the chronic illness answers the scale. | Determine the cohesion within the family about family member perceptions of aspects of integration of the CC or CI into evolving family life and/or determine a sense of the collective family perception of aspects of integration of the Chronic Condition or Chronic Illness into evolving family life. | Family Systems Nursing (FSN), the Reintegration Within Families in the Context of Chronic Illness Model, and measurement theory guided the study. | Family Systems Nursing (Anderson & Tomlinson, 1992; Bell, 2009; Wright & Leahey, 2013) and the Reintegration Within Families in the Context of Chronic Illness Model (Eggenberger, Meiers, Krumwiede, Bliesmer, & Earle, 2011) | 2020 | Meiers, S. J., Eggenberger, S. K., Krumwiede, N. K., & Deppa, B. (2020). Measuring Family Members' Experiences of Integrating Chronic Illness Into Family Life: Preliminary Validity and Reliability of the Family Integration Experience Scale:Chronic Illness (FIES:CI). *Journal of family nursing, 26*(2), 111–125. <https://doi.org/10.1177/1074840720902129> |
